# Crashworthiness Characteristic of Dynamically Expanded Circular Tubes Made of Light Alloys: Experimental and Theoretical Investigation

**DOI:** 10.3390/ma13235332

**Published:** 2020-11-25

**Authors:** Paweł Kaczyński

**Affiliations:** The Department of Plastic Forming, Welding and Metrology, Wrocław University of Science and Technology, Wybrzeże Wyspiańskiego 27, 50-370 Wrocław, Poland; pawel.kaczynski@pwr.edu.pl; Tel.: +48-664-774-877

**Keywords:** aluminum tubes, expansion, precipitation hardening, crashworthiness, energy absorption

## Abstract

The paper presents the process of dynamic expansion of thin-walled pipes made of T6 temper 7075 aluminum alloy. The obtained results were related to the mass of tested elements, which allowed for calculating the specific energy absorption. The registered crushing force was in the range of 600–800 kN/kg. The obtained values were much greater than the crushing force of traditional crash boxes made of steel (80–120 kN/kg) or AZ31 magnesium alloy sheets (150 kN/kg). The analytical model for predicting crushing force depending on the thickness of the pipe wall was developed and proven to be correct by conducting real experiments and FEM simulation. The mathematical model for energy absorption named further as ETMEA (expanding tubes model for energy absorption) was developed based on calculated energy-deflection curves. It was proven that the energy absorption could be easily scaled. In cases where high energy absorption is needed, it can be easily achieved by increasing the thickness, while for lower energy absorption application, the thickness may be reduced and the goal may be achieved by selecting the appropriate length of the profile.

## 1. Introduction

The currently observed trend to reduce the mass of vehicles is determined by several primary factors. The first is the stringent CO_2_ emission target set by the European Union. Currently produced passenger cars must emit no more than 95 g/km of CO_2_. Parliament and the European Council agreed [1] to further reduce CO_2_ emissions by 15% (starting from 2025) and 37.5% (starting from 2030) below currently permitted emission levels. Failure to meet the established requirements will result in the imposition of fines in the amount of 95 EUR for each g/km of target exceedance for each car sold. Due to the lack of disruptive changes in the construction of internal combustion engines, reducing vehicles’ weight is the fundamental way to get closer to the values imposed by law. The Institute for Energy and Transport of the European Commission, in an extensive study [2] on internal combustion cars used in Europe, shows a clear relationship between the weight of the vehicle and the amount of fuel consumed, and thus the amount of emitted harmful substances. The reduction of the vehicle’s weight by 100 kg results in a reduction of fuel consumption by about 0.5 L/100 km.

However, the emission standards are checked at the manufacturers’ level and concern the average emissivity of all offered vehicles of a given brand. Therefore, in order to reduce the average emission of the offered vehicle fleet and sustain the sales of traditional combustion units, a different type of low-emission or zero-emission drive must be introduced. Hence, the dynamic development of electric vehicles is expected. Weiss et al. [3] analyzed a wide range of electric passenger cars (both converted cars and purpose-designed electric cars) and proved that this type of vehicle could also benefit from the use of lightweight materials. A 100 kg reduction in vehicle weight decreases the energy consumption by 0.6 kWh/100 km. The influence of the vehicle weight on its range was also confirmed by Mruzek et al. [4], who simulated the behavior of the experimental electric vehicle named EDISON. He proved that reducing the vehicle’s weight by 10% increases the range by at least 5% and that this amount is slightly dependent on the battery load and the profile of the route traveled. Additionally, by reducing the weight of the vehicle’s supporting structure, it is possible to install additional batteries, and thus further increase the vehicle’s range.

In addition to the need to meet the top-down requirements for exhaust emissions, car manufacturers must also follow customer expectations. In this aspect, it is essential to ensure a sufficiently high level of safety. However, this requirement does not always go hand in hand, and sometimes is even contradictory to the weight reduction of the vehicles produced. The simultaneous achievement of both goals can be met thanks to the use of modern, light alloys, which have an outstanding ratio of dissipated energy to weight—so-called specific energy absorption (SAE). The middle part of the vehicle should be characterized by significant stiffness and low deformation so that the safety cage is not excessively deformed during the crash, providing enough survival space. Thanks to improvements in lightweight alloys’ strength, their use in vehicle bodies is now moving into high gear. Examples include: the body of Audi A8, the space frame of Audi R8, and the all-aluminum alloy monocoque of Jaguar XE and Range Rover Sport [5]. One of the current challenges for carmakers using light alloys is their application to construct the most responsible elements such as the B-pillars that protect passengers during side impacts and the energy-absorbing elements applied in the crumple zones that are the subject of the following study. Their main task is to minimize the forces acting on passengers, which is traditionally achieved by plastic folding of thin-walled elements called crash-boxes.

Among the many alloys of light metals, AZ31B magnesium alloy is one of the most promising materials. Its density equals 1770 kg/m^3^, which is less than ¼ the typical high-strength steel density. Thanks to this, it is possible to obtain high specific energy absorption. An attempt to produce energy-absorbing elements from this alloy was made by Kaczyński et al. [6]. He tested the behavior of 1.2 mm thick tapered elements filled with aluminum foam and without filling. The conducted research proved that the progressive folding of magnesium elements is not possible, which is caused by the excessive amount of energy needed for activation of the sliding planes necessary for successful sheet bending. However, it is possible to use a new mechanism named progressive crushing, which allows for the samples’ predictable behavior. Because cracking and detachment of the material occur during the dynamic crushing, such a solution is unlikely to be used in the automotive industry.

An alternative solution is focused on the use of a high-strength 7075 aluminum alloy. The density of this material equals 2810 kg/m^3^, and its structure can be precipitation hardened in the process of natural or artificial aging to the T6 temper, thanks to which it is possible to obtain the yield point in the range of 500–550 MPa. According to one of the largest consulting companies the Ducker Frontier that has been providing business to business market research in the American market for over 60 years [7], the use of aluminum alloys in the automotive industry will increase by about 25% from 208 kg to about 260 kg per vehicle by 2026. This action will help to meet long-range CO_2_ goals and reduce the mass of BEV platforms that use greater amounts of aluminum sheets, extrusions and castings for mass savings. The disadvantage of the discussed alloy is the low elongation at break equal to about 0.2. The use of a plastic folding mechanism for energy absorption involves the occurrence of the strain equal to 0.5 and more. Therefore, there is a justified concern that using aluminum sheets for the production of crash-boxes may result in a local loss of material stability.

In the following paper, a different mechanism of energy absorption is proposed, i.e., tube expansion. The process consists in forcing a cylinder into a thin-walled tube, so that the outer diameter of the cylinder is greater than the inner diameter of the tube. The size of the interference is selected in such a way that permanent plastic deformation of the pipe occurs.

The expansion of thin-walled tubes with a rigid cylindrical-conical die was investigated by Min et al. [8], who distinguished three primary modes of deformation (T-C mode, W-C mode, and W-CC model) depending on the ratio of the wall thickness of the pipe to its diameter and the apex angle of the expanding element. The correctness of the created models was proved by carrying out an experiment of static expansion of pipes with a thickness of 1, 2, 3, and 5 mm. The experimental data were convergent with the analytical models.

The attempt to improve the energy absorption characteristics of circular metal tubes subjected to axial loading was made by Salehghaffari et al. [9]. The solution presented in the paper consists of the use of the 25–90 mm high, 2 mm thick expanding steel ring, which was pressed into the upper part of the tube (2.5 mm thick, 200–350 mm long, internal diameter equal to 70 mm). Next, the assembly was quasi-statically compressed by flat plates. The described solution did not affect the compressive force’s value, but it ensured the correct deformation mode. The folding was present only on that side of the pipe, where no ring was installed. It is worth noting that an unspecified aluminum alloy of insufficient strength (*R*_e_ = 175 MPa, *R*_m_ = 200 MPa, *A* = 0.1) was used to produce the pipes, which could be the cause of their folding on both sides.

Static expansion tests of 5A06 aluminum alloy (medium strength alloy, *R*_e_ = 250 MPa, *R*_m_ = 350 MPa, *A* = 0.2) pipes with thicknesses from 1 to 5 mm were undertaken by Jialing et al. [10]. Conical-cylindrical, rigid expanding elements with a different conical angle of 5°, 10°, 15° and 20° were used. Additionally, the conducted research was supplemented with numerical simulations conducted in the MSC MARC system. Two stages of the deformation process have been distinguished. In the first one, there were significant oscillations of force, which was related to establishing the mutual position of both elements. Next, after forcing in the expanding element to a depth of 30 mm, the force value stabilized. It was noticed that increasing the apex angle of the conical part of the expanding element result in a significant increase in the compressive force. However, this phenomenon occurs only for the pipe’s wall thicknesses greater than 3 mm. Moreover, it was observed that the use of MoS_2_ solid lubricant allows the compressive force to be reduced approximately twice and to obtain an oscillation-free force-displacement signal in the second phase of expansion.

Another study concerning expanding circular tubes in order to dissipate the kinetic energy of the dynamic impacts through plastic deformation and friction was done by Yan et al. [11]. The paper presents a theoretical model that takes into consideration shear deformation and the results of FEM numerical modeling. Both approaches were in agreement. It was proven that forcing the expanding element with an external diameter of 84 mm into a steel pipe with an internal diameter of 72 mm has been proven to be an effective method of absorbing energy. The testing velocity was set to 6.39 m/s. The linear influence of the friction coefficient on the compressive force that equals 337.5 kN for *μ* = 0 and 668.7 kN for μ = 0.3 was determined. A particular inconvenience of the work is the lack of actual experiments.

One of the main disadvantages of the discussed process is the need to use a heavy, volume expanding element, which is usually made of high-strength steel. An attempt to reduce its mass was made by Shakeri et al. [12], who replaced a rigid die with a heat-hardened steel pipe made of steel with the yield strength *R*_e_ = 235 MPa and the deformation at break 0.19. The presented theoretical model of the expansion was consistent with the results of the tests and numeric simulations, which proves the possibility of optimizing the expansion technology.

Another version of the described solution is a stainless cylindrical-conical die used by Jian et al. [13]. At first, the pipe is expanded by the cylindrical part of the die that is forced into it. Then, the tube begins to slide down the conical part of the die, which results in further expansion of the pipe. Due to the fact that eight symmetrically spaced cuts (cracks initiators) were made to a depth of 5 mm along the central axis of the pipe, further penetration results in cracking of the pipe, so that sheet strips were formed and then curled. The presented mechanism of deformation and cracking allowed increasing the maximum force by about 95% in relation to the force needed to expand the pipe by the cylindrical part of the sleeve. However, it should be noted that it is highly probable that an analogous increase in force could be obtained by increasing the interference of both parts, which could be achieved by increasing the diameter of the cylindrical part of the die. Moreover, due to the material’s cracking, which cannot be fully controlled in dynamic conditions, the discussed process cannot be used in the automotive industry.

Another interesting solution was tested by M.M. AbdElwahab [14], who presented a design based on circular stepped tubes based on free inversion. The specimen consists of two pipes that are welded together. The lower one is tapering upwards and the upper one is tapering downwards. Depending on both pipes’ convergence angles, as the crushing continues, it is possible to expand the lower pipe (in addition to inversion). The main achievement includes a 16% reduction of the specimen mass and 31% higher energy absorption in comparison to the design allowing for a sole inversion of the pipes.

This work focuses on the possibility of using the high-strength T6 temper 7075 aluminum alloy for the construction of expanded pipes under conditions of dynamic loading. Additionally, an attempt was made to determine the coefficient of friction between steel and aluminum. For this purpose, FEM numerical methods, analytical calculations and experimental tests were used.

## 2. Materials and Methods

Evaluation of the energy absorption properties of thin-walled tubes made of aluminum and steel will allow estimating the possibility of using them in the automotive industry, in particular as the replacement for traditional crash-boxes. The conducted tests should also answer the question of which of the material thickness is preferable. The thin-walled specimens were prepared with the use of two different materials, namely:

- S355JR: hot rolled, non-alloy, structural steel of a higher quality and strength. This type of material is characterized by fine-grained microstructure, which results in the minimum yield strength equal to *R*_emin_ = 355 MPa and tensile strength in the range of *R*_m_ = 470–630 MPa. The minimum percentage elongation after fracture is equal to 22%. The maximum amount of the following elements may be found in the chemical composition: 0.24 C, 0.55 Si, 1.6 Mn, 0.035 P, 0.035 S, 0.012 N, 0.55 Cu [15].
- T6 temper 7075 aluminum alloy: This is age-hardenable, precipitation hardened 7000 series aluminum alloy. Thanks to the solution heat treatment (SHT) process and subsequent artificial aging, the material can gain high strength. The material’s initial hardness was equal to135–150 HBW30, which results in the yield strength in the range of *R*_emin_ = 450–510 MPa.

**Figure 1:**
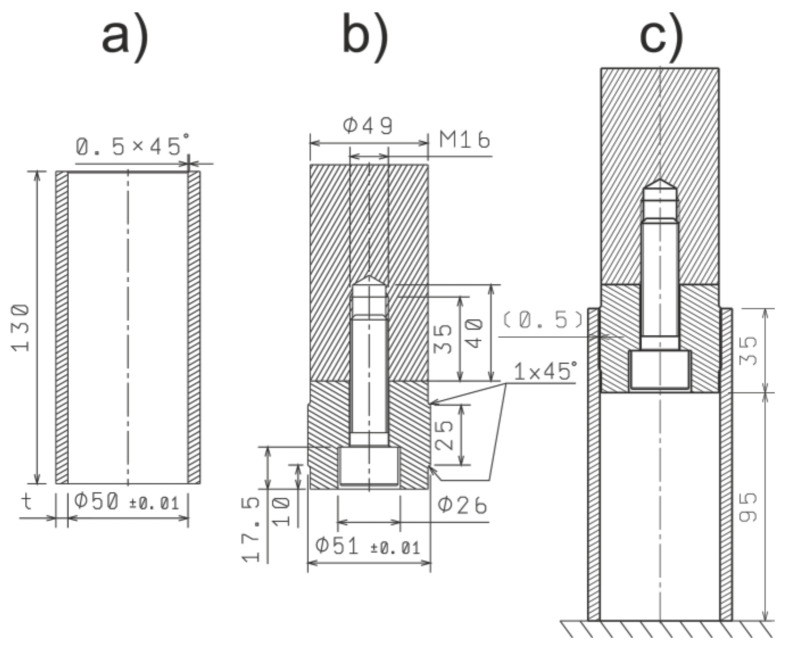
The dimensions of the specimens. Dimensions in millimeters: (**a**) tube; (**b**) expanding element; (**c**) assembly.

The specimens were manufactured from commercially available thick pipe (S355) and a rod (T6 temper 7075 aluminum alloy) to its final shape (Figure 1a), which is presented below. The inner and outer surfaces were straight turned, while both ends were faced in order to obtain flat and parallel surfaces. The final inner diameter was equal to ∅50 mm, while the thickness *t* was equal to 2.0, 2.5, 3.0, 3.5, or 4.0 mm. The length of the specimen was equal to 130 mm.

The element used for expanding the specimens consisted of two parts screwed together, which is presented in Figure 1b. The main part that causes expansion of the pipe was made of 40H steel grade hardened to 49 HRC, while the extension was made of S355 constructional steel. The external dimensions of the expanding element (51 mm) and the pipe’s internal dimension (50 mm) were selected in such a way that there is 1.0 mm of interference.

Before the dynamic testing, the expanding element was pressed into the tube to a depth of 35 mm by means of the manual hydraulic press, which resulted in completely hiding the expanding element’s part of a bigger diameter inside the tube, which is depicted in Figure 1c. The plan of the test, as well as the basic material properties, was presented in Table 1.

### 2.1. Quasi-Static Tests

In order to evaluate the energy-absorption abilities of the specimens, two aluminum tubes and two steel tubes were tested in quasi-static conditions prior to dynamic impacts. The tests were carried out by centering the specimen with the expanding element pushed in beforehand (Figure 1c) on a flat, lower plate attached to the transverse of the universal testing machine (Zwick/Roell ZMART.PRO, Zwick Roell, Ulm, Germany). Then, the specimens were pushed against the flat upper plate mounted on a ball-and-socket joint to the force sensor with a measuring range of up to 100 kN. The constant traverse speed of 0.2 mm/s was applied during the test. The compressive force and the crossbar travel were accordingly measured, which allowed constructing force-displacement and energy-displacement curves.

### 2.2. Dynamic Tests

**Figure 2:**
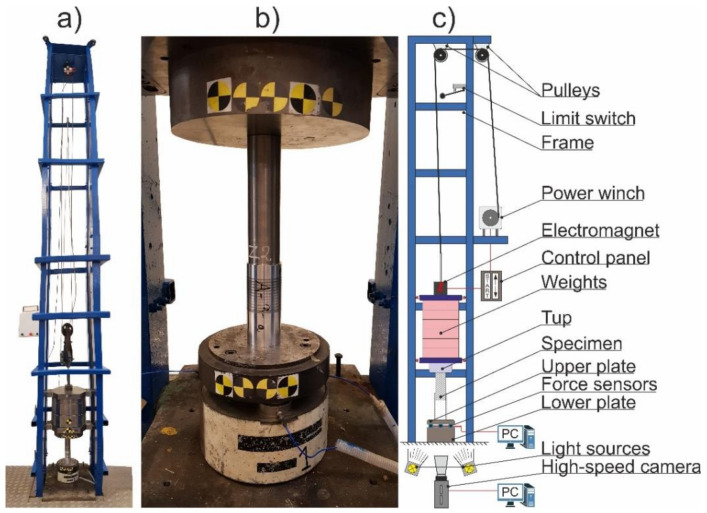
Gravity drop hammer: (**a**) photograph; (**b**) position of the specimens; (**c**) scheme.

The dynamic crushing experiments were conducted with the use of a gravity drop hammer, whose design was developed by the author. The device consisted of a metal frame made of channel bars to which brass guides were attached. The free-falling, 227.2 kg mass that consists of the weights and the hardened tup was moving along the guides. The impact height was set to 2.0 m, which resulted in an impact velocity of 6.26 m/s and potential energy of moving parts equal to about 4.46 kJ. The crushing force was measured utilizing three high-frequency, piezoelectric force sensors (PCB Piezotronics, New York, NY, USA) that were located under the metal anvil on which the specimens were centered. The device, the specimens’ position, and the test stand’s scheme are depicted in Figure 2. The sampling frequency of the force sensors was equal to 100 kHz. Each series consisted of a minimum of 2 samples of the same thickness.

The tubes’ dynamic crushing was additionally recorded by a V12 Phantom (Vision Research, Wayne, NJ, USA) high-speed camera. Due to the oscillation of the test stand, this is one of the most reliable registration methods allowing for fast and error-free measurement of the element’s displacement. The test stand was illuminated with a high-intensity light and the positions of fiducial markers placed beforehand on the and anvil were recorded with the framerate of 15 kHz. Next, the markers’ exact positions were determined by means of TEMA motion analysis software (Image Systems Motion Analysis, Linköping, Sweden). Next, in order to provide uniform sampling frequency, the camera signal was interpolated with FlexPro analysis software (FlexPro 7.0, Weisang GmbH, St. Ingbert, Germany). After this treatment, the force-displacement and energy-displacement graphs were further analyzed with standard engineering programs like MS Excel (Microsoft, Redmond, WA, USA).

### 2.3. FEM Modeling

**Figure 3:**
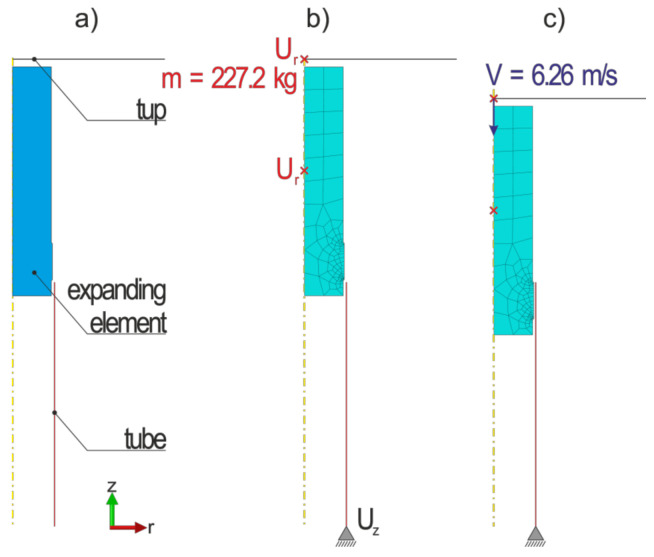
FEM computational model: (**a**) geometric model; (**b**) discrete model; (**c**) position of the element before the proper dynamic tests.

In order to enable accurate FEM modeling of the expansion of thin-walled tubes, an axisymmetric geometric model was prepared. Its geometry corresponded to the dimensions of the actual samples. The model consisted of three parts: deformable tube, rigid tup and rigid expanding element, as depicted in Figure 3a. The tube and the tup were discretized (Figure 3b) using a 2-node linear, axisymmetric thin shell elements (SAX1), while the expanding element was discretized using a 4-node bilinear, axisymmetric, quadrilateral, reduced integration, hourglass control elements (CAX4R). The boundary conditions of the model are presented in Figure 3b,c, where U_z_ and U_r_ mean restraining the translation along *z* and *r* directions. In order to minimize the slip-stick numerical phenomenon, the size of the discrete elements of the tube was set to 0.4 mm. In order to reduce the calculation time, the rest of the rigid elements were discretized using elements’ size in the range of 1–10 mm. The surface-to-surface contact type was applied and the penalty contact method was used to describe mechanical constraint formulation. In order to track which part of the master surface is in contact with each slave node, the finite-sliding option was applied. The adopted flow rule was “associated plastic flow”. Therefore, as the material yields, the inelastic deformation rate is in the direction of the normal to the yield surface (the plastic deformation is volume invariant). This assumption is generally acceptable for most calculations with metals.

The expanding element was pushed quasi-statically inside the tube before the proper expanding test in order to reach the initial position depicted in Figure 3c. It was accomplished by inducing displacement of the expanding element’s reference point. In the second step, all of the forces are released, except for the tube’s support. Next, the 227.2 kg tup moving freely along z-direction hit the expanding element.

In order to validate the value of the calculated friction coefficient, the crushing force and the displacement of the expanding element were registered during numerical simulation carried out for a broad spectrum of friction coefficient in the range of (0.1–0.5) and for each tested material thickness (2.0, 2.5, 3.0, 3.5, and 4.0 mm). The simulations were conducted in ABAQUS software 6.14-4.

## 3. Results and Discussion

### 3.1. Quasi-Static Tests

**Figure 4:**
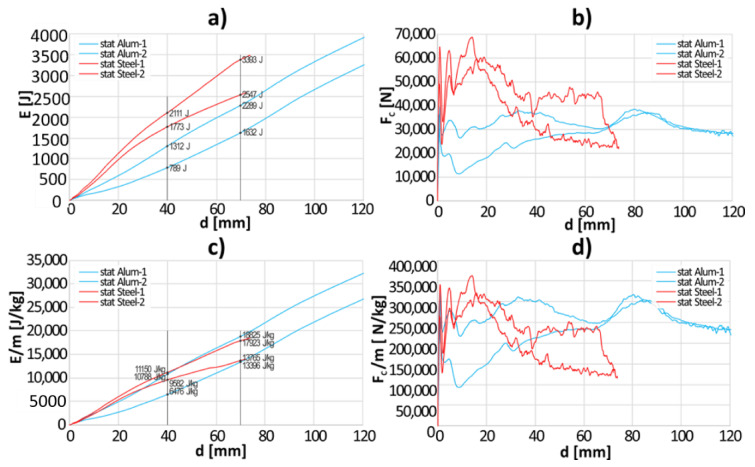
The results of quasi-static compression tests: (**a**) *E* = f(d); (**b**) *F_C_* = f(d); (**c**) *E*/m = f(d); (**d**) *F_C_*/m = f(d).

Two aluminum tubes and two steel tubes were tested in quasi-static conditions. The crushing force and the energy curves were presented as a function of the expanding element’s displacement (Figure 4a,b). Additionally, both values were divided by the weight of that part of the sample that was deformed during expansion, which is presented in Figure 4c,d.

The absorbed energy as well as specific energy were analyzed for the displacement of the expanding element, equal to 40 and 70 mm. In the case of steel tubes, the average energy absorbed after 40 and 70 mm displacement was equal, respectively, to *E*_40_ = 1942 J and *E*_70_ = 2970 J. In the case of tubes made of aluminum alloy the values were lower by about 34%–46% and amounted, respectively, to *E*_40_ = 1050 J and *E*_70_ = 1960 J.

Next, the specific energy absorption was analyzed for the same displacement of the expanding element. The result for the steel tubes are as follows: *E*_40_/m = 10,366 J/kg and *E*_70_/m = 15,844 J/kg. In the case of the tubes made of aluminum alloy, the values amounted respectively to *E_40_*/m = 8632 J/kg and *E*_70_/m = 16,110 J/kg. When the specimens’ weight was taken into consideration, it turned out that the aluminum tubes performed similarly to the steel tubes, which confirmed the purposefulness of conducting the dynamic tests.

### 3.2. Dynamic Tests

**Figure 5:**
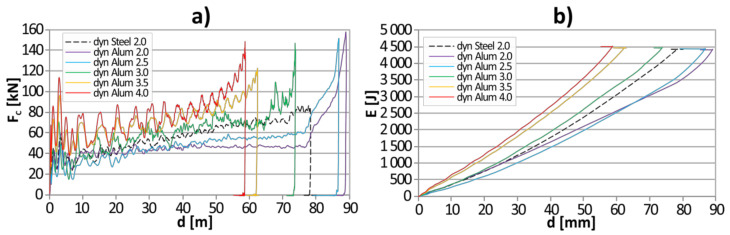
The results of dynamic compression tests: (**a**) *E* = f(d); (**b**) *F_C_* = f(d).

The results of dynamic crushing tests were presented in the form of force-displacement curves (Figure 5a). Additionally, the amount of energy absorbed during the tubes’ expansion was calculated and presented in Figure 5b. Due to the overlapping of the results in each series, the mean curves are presented.

The observed oscillations were caused by several main factors. First of all, the expanding element is only in periodic contact with the falling mass. It bounces off the falling mass, which can be seen in the videos recorded with a high-speed camera. This phenomenon is particularly intense at the beginning of the process. Then the amplitude of the reflections is getting smaller. Additionally, the slip-stick and periodical changes of the friction coefficient in individual mutually contacting regions of the falling mass and the expanding element may occur. It is also worthy notifying that elastic waves, both longitudinal and transverse, propagate in the sample and the tooling so that some of them are recorded as noise after multiple reflections.

The first conclusion is that the thickness of the wall of aluminum tubes has a significant influence on the specimen crashworthiness, i.e., on the crushing force and the displacement of the expanding element. The maximum displacement (d) of the expanding element reached the values of 89.0, 86.8, 73.8, 62.4, and 58.8 mm, which resulted from the consecutive increase of the wall thickness (*t*) to the values of 2.0, 2.5, 3.0, 3.5, and 4.0 mm. The relation between both parameters may be described by the equation d = −17*t* + 125, which is characterized by the coefficient of determination *R*^2^ = 0.95. This means that an increase in wall thickness by 1 mm results in a reduction in deflection by 17 mm on average. The maximum relative percentage error between calculated and measured deflection is equal to 5%. It should be underlined that the formula stays valid for the thickness range from 2.0 to 4.0 mm. The formula can also be applied to values outside this range, but an increase in inaccuracy should be expected while moving away from the applicability range.

Additionally, the average value of the crushing force of aluminum tubes was calculated for the deflection range of 20–40 mm. It is equal to 44.8 kN (*t* = 2.0 mm), 46.2 kN (*t* = 2.5 kN), 62.1 kN (*t* = 3.0 mm), 72.1 kN (*t* = 3.5 mm), and 78.1 kN (*t* = 4.0 mm). In order to compare the calculated values with the theoretical crushing force, the circumferential deformation of the expanded pipes was determined based on the radius of the tube before the expansion (*r*_0_) and after it (*r*_1_) as:

(1) $$\varepsilon =\frac{∆l}{l_{0}}=\frac{2\pi \left[\left(r_{1}+\frac{t}{2}\right)-\left(r_{0}+\frac{t}{2}\right)\right]}{2\pi \left(r_{0}+\frac{t}{2}\right)}=\frac{r_{1}-r_{0}}{r_{0}+\frac{t}{2}}.$$

In the case of selected dimensions of the tube and the expanding element, the theoretical circumferential strain should be, depending on wall thickness, in the range of *ε* = 0.0185–0.0192. In the case of thin-walled elements, the wall of the tube may be treated as a flat surface and according to the Young-Laplace equation, the circumferential stress (*σ_c_*) created by internal pressure (*p*) may be calculated according to [16,17] as:

(2) $$\sigma_{C}=\frac{p\times \left(r_{1}+\frac{t}{2}\right)}{t}.$$

On the basis of Equation (2) the total amount of normal force (*F_N_*) exerted on a surface (*A*) of the expanding element’s part of a wider diameter can be calculated as:

(3) $$F_{N}=p\times A=\frac{\sigma_{c}\cdot t}{\left(r_{1}+\frac{t}{2}\right)}\times 25\times 2\pi r_{1}=50\frac{\sigma_{c}\cdot t\cdot \pi \cdot r_{1}}{\left(r_{1}+\frac{t}{2}\right)}\;\left[N\right].$$

This leads to the theoretical calculation of the crushing force *F_C_* needed to force in the expanding element inside the tube. The force depends on the friction coefficient (*μ*) between aluminum and steel and may be calculated using the Equation (4)

(4) $$F_{C}=F_{N}\times \mu =50\frac{\sigma_{c}t\cdot \pi \cdot r_{1}\cdot \mu }{\left(r_{1}+\frac{t}{2}\right)}\;\left[N\right].$$

**Figure 6:**
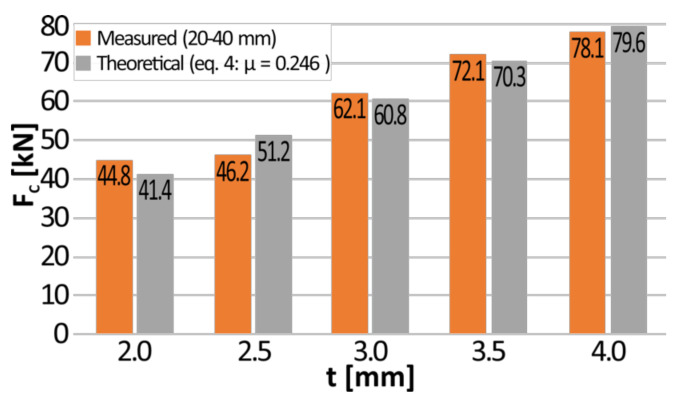
The comparison of calculated and measured crushing force.

The value of the friction coefficient was adjusted in such a way that the difference between theoretical crushing force *F_C_* calculated on the basis of Equation (4) and the average value of the crushing force measured in the interval from 20 mm to 40 mm was minimized for each of the tested thicknesses of the tubes. The average calculated value of the friction coefficient between T6 temper 7075 aluminum alloy and 40H steel equals *μ* = 0.246 and the comparison of calculated and measured crushing force is presented in Figure 6. The maximum relative error between the theoretical values and the measured ones equals to 11%. It should be underlined that the friction coefficient may vary during the process due to changes in the adhesion.

The calculated friction coefficient is in agreement with the results presented by Sanborn et al. [18], who determined the friction coefficient between 4340 steel and T6 temper 7075 aluminum alloy. It is worth mentioning that the authors have tested samples at a velocity of 8.32 m/s, which about 30% higher than the velocities presented in the following paper. Additionally, a Kolsky tension bar with dedicated friction grip fixtures was used, which is a well-known, reliable and repeatable way to measure the friction coefficient between metallic materials. In the case of smooth finish of both surfaces, the friction coefficient amounted, respectively, to 0.23 ± 0.02 (static) and 0.16 ± 0.01 (kinetic), while rough finish resulted in about 25%–30% higher values of respectively 0.30 ± 0.03 (static) and 0.20 ± 0.02 (kinetic).

Next, on the basis of calculated energy-deflection curves presented in Figure 5b, the mathematical model for energy absorption named ETMEA (expanding tubes model for energy absorption) was developed. In order to check the correctness of the model, additional statistical parameters were checked. The coefficient of multiple determination amounted to *R*^2^ = 0.978 and the standard error of the estimate was equal to σ = 175. The ETMEA model was set as:

(5) $$E\left(d,t\right)=A\times d^{B}\times C^{t},$$

where independent variables were denoted as:

*d*—expanding element deflection (mm),

*t*—tube thickness (mm).

The values of dimensionless coefficients of A, B, and C were determined during nonlinear regression analysis. They are presented in Table 2.

**Figure 7:**
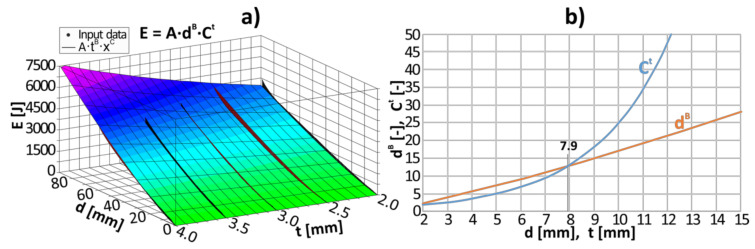
The energy absorption model ETMEA: (**a**) graphical representation; (**b**) the influence of crucial factors on energy absorption.

The graphical representation of the ETMEA model is presented in Figure 7a in the form of a three-dimensional surface that fits the best to the input data. The influence of both parameters, i.e., thickness (*t*) of the tube and the deflection (*d*) of the on the total amount of absorbed energy was compared and the results were presented in Figure 7b. The influence of the deflection may be described by the power function. Since constant B = 1.232, which is close to 1, the influence of the deflection on the absorbed energy is almost linear in the range from 0 mm to 100 mm. On the other hand, the influence of the thickness of the tube is exponential. The base is greater than 1 and equal to C = 1.380, which results in exponential growth. When both factors (C*^t^*, *d*^B^) are compared, the following conclusion may be drawn. Starting from the value of about 8, the influence of the thickness is much more significant than the influence of the deflection. Because the ETMEA model has both power and exponential element, the energy absorption can be easily scaled. In cases where high energy absorption is needed, it can be easily achieved by increasing the thickness, while for lower energy absorption application, the thickness may be reduced and the goal may be achieved by selecting the appropriate length of the profile.

**Figure 8:**
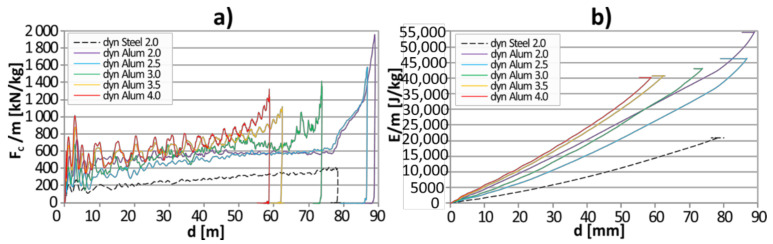
The results of dynamic compression tests: (**a**) *E*/m = f(d); (**b**) *F_C_*/m = f(d).

Additionally, both crushing force and the amount of absorbed energy were divided by the weight of that part of the sample that was deformed during expansion, which is presented in Figure 8a,b. This action clearly revealed the potential behind the tested energy absorption mechanism. Independently of the tube’s thickness, the curves representing the axial crushing force per consumed mass (Figure 8a) for aluminum specimens were at least 1.8 times higher than the corresponding curve representing steel tube. The specific energy absorption (SEA) curves for all aluminum tubes were1.8–3.0 times steeper than the curve representing SEA of steel tube.

When compared with competitive solutions, the expanded elements are characterized by top crashworthiness. As depicted in Figure 8, the tested elements can dissipate as much as 20,000–30,000 J/kg and the registered crushing force is in the range of 600–800 kN/kg (for a deflection of 50 mm). The typical, thin-walled crash-boxes used in the automotive industry tested by Gronostajski et al. [19] can absorb no more than 7000 J/kg and their crushing force is in the range of 80–150 kN/kg (for the same deflection). The exact values equal respectively to 7000 J/kg and 150 kN/kg (for 1.8 mm thick AZ31 + Al foam), 6250 J/kg and 120 kN/kg (for 1.5 mm thick HC380) as well as 3750 J/kg and 80 kN/kg (for 1.5 mm thick DC04).

In order to maintain the reliability of results, it is necessary to consider the construction of both types of elements. While a traditional crash-box can be mounted directly between the vehicle’s crossbeam and the longitudinal, expanded elements need additional component, which is pressed into the pipe. This element is usually a solid steel element of considerable weight, which will significantly reduce the presented solution’s efficiency. For this reason, it is recommended to use the presented mechanism of energy absorption in applications where the weight of the structure is not an essential factor or to modify the presented solution so as to replace the expanding element with a second pipe that will be pressed into the outer pipe. As a result, both pipes will undergo plastic deformation. This should provide only a slight reduction of the compressive force or the amount of absorbed energy. The described solution is the direction of further research and requires conducting detailed analyzes.

### 3.3. FEM Modeling

**Figure 9:**
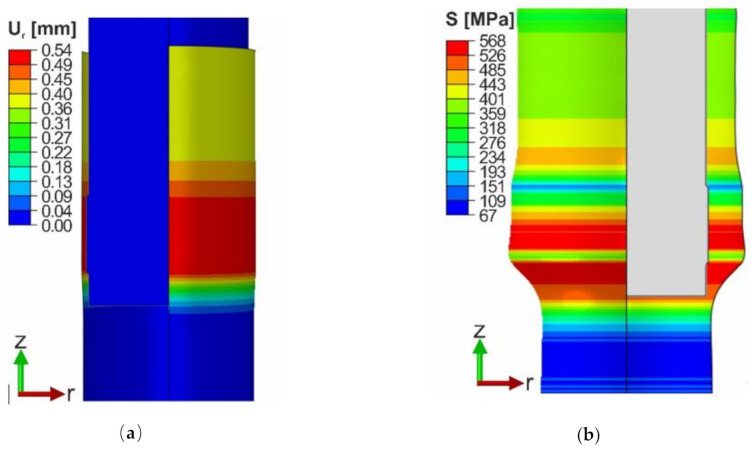
The contour lines of r-displacement (FEM): (**a**) Displacement; (**b**) Von-Mises Stress.

In the first step, the constant friction coefficient equal to *μ* = 0.246 was set. The tube’s radius increases by 0.5 mm in the area of the greatest diameter of the expanding element. As the process progress, when the expanding element goes deeper into the material, the tube shrinks. Due to permanent plastic deformation, the radius is reduced to a value that is bigger by about 0.38 mm than the initial radius (Figure 9a). The stress distribution is depicted in Figure 9b. The material is expanded by the lower edge of the wider part of the expanding element. Additionally, due to the material’s bending, the stress heterogeneity can be observed in the areas where the diameter changes.

**Figure 10:**
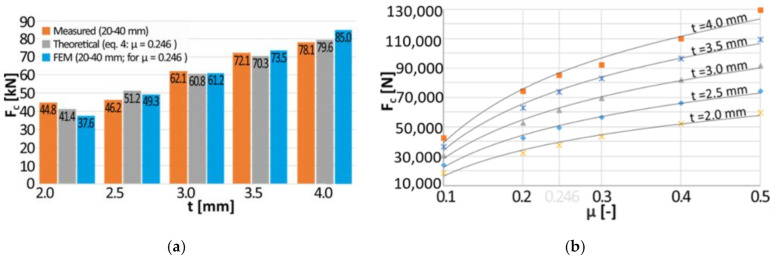
The results of the simulation of expanding tubes: (**a**) *E*/m = f(d); (**b**) *F_C_*/m = f(d).

Based on the force and displacement determined during the simulation, an average crushing force in the deflection range of 20–40 mm was calculated. The results were compared with the theoretical crushing force *F_C_* calculated on the basis of Equation (4) and the average value of the crushing force measured during the experiment. The results are depicted in Figure 10a. The FEM models were in agreement with theoretical force as well as with values registered during the experiment. The maximum relative error between the theoretical values of the crushing force and FEM based predictions is equal to 9%.

The value of the average crushing force was also determined for friction coefficient in the range of (0.1–0.5) and for material thickness in the range of 2.0–4.0 mm. The results are presented in Figure 10b. The points for each of the evaluated thicknesses were approximated using logarithmic function *Fc* = a × ln(μ) + b. The values of (a) and (b) coefficients along with their 90% confidence intervals, as well as the coefficient of multiple determination (*R*^2^) were presented in Table 3.

It is worth noticing that the friction coefficient has a crucial impact on the crushing force level. Independently of the tested thickness changing the coefficient of friction from 0.1 to 0.5 allows tripling the value of the compressive force, and thus the amount of absorbed energy.

## 4. Conclusions

Summarizing, the conclusions drawn from the conducted research can be recapitulated as follows:

1. The maximum displacement (d) of the expanding element is the function of the tube’s thickness (*t*) and may be described by the equation *d* = −17*t* + 125. This allows tailoring the geometry of the energy-absorbing element in order to obtain the desired maximum deflection. The maximum relative percentage error between calculated and measured deflection is equal to 5% in the applicability range from 2.0 to 4.0 mm.
2. It should be underlined that the friction coefficient may vary during the process due to changes in the adhesion. The average friction coefficient between the tube made of T6 temper aluminum alloy and the expanding element made of 40H steel grade that was forced into the tube at a rate of 6.26 m/s was calculated to be equal to *μ* = 0.246. This observation was found to be in good agreement with the theoretical predictions, FEM models and the tests conducted by Sanborn et al. [18]
3. The expanding tubes model for energy absorption (ETMEA) was developed. The amount of absorbed energy was described as E(*d*,*t*) = A × *d*^B^ × C^t^. Because there are both power and exponential elements in the model, the energy absorption can be easily scaled. For high energy absorption, the thickness’s increase is advised, while for lower energy absorption application, the thickness may be reduced and the goal may be achieved by selecting the appropriate length of the profile.
4. The tested elements show great potential to be used as energy-absorbing elements. The axial crushing force divided mass of the expanded part of the tube was at least 1.8 times higher than the corresponding curve representing steel tube. The specific energy absorption (SEA) curves for all of the aluminum tubes were 1.8–3.0 times steeper than the curve representing SEA of steel tube. The described conclusion is valid independently of the thickness of the tube.

## Figures and Tables

**Table 1:** Basic properties of tested material and the test plan.

Material Name (-) Density (kg/m^3^) Young’s Modulus (GPa) Re (MPa) Poisson’s Ratio (-)	Testing Condition (-)	Crushing Velocity (mm/s)	Thickness (mm)
Steel 7860 210 406 0.3	Quasi-static	0.2	2.0
	Dynamic	4300	2.0
Aluminum 2810 72 512 0.33	Quasi-static	0.2	2.0
	Dynamic	4300	2.0, 2.5, 3.0, 3.5, 4.0

**Table 2:** Values of the parameters of ETMEA model.

Parameter	Value	90% (+/−)	95% (+/−)	99% (+/−)
A	8.237	0.135	0.161	0.212
B	1.232	0.003	0.004	0.005
C	1.380	0.002	0.002	0.003

**Table 3:** Values of the parameters of ETMEA model.

Thickness (mm)	a (−)	b (−)	*R* ^2^	a90% (+/−)	b90% (+/−)
2.0	25,480	75,099	0.984	3440	4990
2.5	31,406	94,658	0.993	2812	4079
3.0	38,336	116,466	0.993	3295	4780
3.5	45,253	138,240	0.994	3774	5476
4.0	52,424	159,788	0.984	7189	10,430
